# Supplementary material for: Probabilistic Modeling of Antibody Kinetics Post Infection and Vaccination: A Markov Chain Approach
Source: ArXiv. 2025 Jul 14:arXiv:2507.10793v1. Preprint. [Version 1] (PMC12288654)
Supplement: Supplementary file 1 [file NIHPP2507.10793v1-supplement-1.pdf]

A Additional time-inhomogeneous two event model details

To better illustrate the time-inhomogeneous two event transition matrix S given by (11), we explicitly calculate the probability of a person residing in a particular class after five time steps using the product of transition matrices:

$$
\begin{aligned}
&S(4)S(3)S(2)S(2)S(1)S(0)\boldsymbol{e}_1 \\
&= \begin{bmatrix}
s_N(4)s_N(3)s_N(2)s_N(1)s_N(0) \\
s_{\mathcal{I}N}(4)s_N(3)s_N(2)s_N(1)s_N(0) \\
s_{\mathcal{V}N}(4)s_N(3)s_N(2)s_N(1)s_N(0) \\
s_{\mathcal{I}N}(3)s_N(2)s_N(1)s_N(0) + s_{\mathcal{I}'}(4)\left\{s_{\mathcal{I}N}(2)s_N(1)s_N(0) + s_{\mathcal{I}'}(3)[s_{\mathcal{I}N}(1)s_N(0) + s_{\mathcal{I}'}(2)s_{\mathcal{I}N}(0)]\right\} \\
s_{\mathcal{V}N}(3)s_N(2)s_N(1)s_N(0) + s_{\mathcal{V}'}(4)\left\{s_{\mathcal{V}N}(2)s_N(1)s_N(0) + s_{\mathcal{V}'}(3)[s_{\mathcal{V}N}(1)s_N(0) + s_{\mathcal{V}'}(2)s_{\mathcal{V}N}(0)]\right\} \\
s_{\mathcal{I}\mathcal{I}'}(4)\left\{s_{\mathcal{I}N}(2)s_N(1)s_N(0) + s_{\mathcal{I}'}(3)[s_{\mathcal{I}N}(1)s_N(0) + s_{\mathcal{I}'}(2)s_{\mathcal{I}N}(0)]\right\} \\
s_{\mathcal{V}\mathcal{I}'}(4)\left\{s_{\mathcal{I}N}(2)s_N(1)s_N(0) + s_{\mathcal{I}'}(3)[s_{\mathcal{I}N}(1)s_N(0) + s_{\mathcal{I}'}(2)s_{\mathcal{I}N}(0)]\right\} \\
s_{\mathcal{I}\mathcal{V}'}(4)\left\{s_{\mathcal{V}N}(2)s_N(1)s_N(0) + s_{\mathcal{V}'}(3)[s_{\mathcal{V}N}(1)s_N(0) + s_{\mathcal{V}'}(2)s_{\mathcal{V}N}(0)]\right\} \\
s_{\mathcal{V}\mathcal{V}'}(4)\left\{s_{\mathcal{V}N}(2)s_N(1)s_N(0) + s_{\mathcal{V}'}(3)[s_{\mathcal{V}N}(1)s_N(0) + s_{\mathcal{V}'}(2)s_{\mathcal{V}N}(0)]\right\} \\
s_{\mathcal{I}\mathcal{I}'}(3)[s_{\mathcal{I}N}(1)s_N(0) + s_{\mathcal{I}'}(2)s_{\mathcal{I}N}(0)] + s_{\mathcal{I}\mathcal{I}'}(2)s_{\mathcal{I}N}(0) \\
s_{\mathcal{V}\mathcal{I}'}(3)[s_{\mathcal{I}N}(1)s_N(0) + s_{\mathcal{I}'}(2)s_{\mathcal{I}N}(0)] + s_{\mathcal{V}\mathcal{I}'}(2)s_{\mathcal{I}N}(0) \\
s_{\mathcal{I}\mathcal{V}'}(3)[s_{\mathcal{V}N}(1)s_N(0) + s_{\mathcal{V}'}(2)s_{\mathcal{V}N}(0)] + s_{\mathcal{I}\mathcal{V}'}(2)s_{\mathcal{V}N}(0) \\
s_{\mathcal{V}\mathcal{V}'}(3)[s_{\mathcal{V}N}(1)s_N(0) + s_{\mathcal{V}'}(2)s_{\mathcal{V}N}(0)] + s_{\mathcal{V}\mathcal{V}'}(2)s_{\mathcal{V}N}(0)
\end{bmatrix}
\end{aligned}
\tag{A1}
$$

We interpret a few entries of (A1) as follows. The first entry, $\text{Prob}(X_4 = N)$, computes the probability that one stays naïve through time step 5; the second entry, $\text{Prob}(X_4 = \mathcal{I})$, gives the probability that one did not become infected on time steps 1, 2, 3, or 4, but did become infected on time step 5. The fourth entry, $\text{Prob}(X_4 = \mathcal{I}')$, is the probability that one did not become infected on time steps 1, 2, or 3, but did become infected on time step 4 and is considered previously infected on time step 5 plus the probability that one became infected on a previous time step (computed by adding the individual event probabilities together since they are disjoint), was then considered previously infected, and remained previously infected on subsequent time steps including time step 5. The sixth entry, $\text{Prob}(X_4 = \mathcal{I}\mathcal{I}')$, is the probability that one became infected on time steps 1, 2, or 3, then remained previously infected, then becomes newly infected on the fifth time step (indicated by the term $s_{\mathcal{I}\mathcal{I}'}(4)$). The quantity in curly braces appears in the fourth entry, too, denoting infection before time step 4. The tenth entry, $\text{Prob}(X_4 = \mathcal{I}'\mathcal{I}')$, is the probability that one became infected on time step 1 or 2 and becomes newly reinfected on time step 4 and is considered previously reinfected on time step 5, plus the probability that one became infected on time step 1, reinfected at the next possible opportunity (time step 3), and then is considered previously infected thereafter. We confirm that all possible trajectories to $N, \mathcal{I}, \mathcal{I}', \mathcal{I}\mathcal{I}'$, and $\mathcal{I}'\mathcal{I}'$ on time step 5 are considered. Parallel interpretations hold for quantities involving $\mathcal{V}, \mathcal{V}'$, and cross events of infection after vaccination or vice versa.

Recalling from (12) that multi-step transitions can be represented by repeated multiplications of the matrix S, we generalize the above concepts to an arbitrary number of time steps $T+1$:

$$
\begin{bmatrix}
\text{Prob}(X_T = N) \\
\text{Prob}(X_T = \mathcal{I}) \\
\text{Prob}(X_T = \mathcal{V}) \\
\text{Prob}(X_T = \mathcal{I}') \\
\text{Prob}(X_T = \mathcal{V}') \\
\text{Prob}(X_T = \mathcal{I}\mathcal{I}') \\
\text{Prob}(X_T = \mathcal{V}\mathcal{I}') \\
\text{Prob}(X_T = \mathcal{I}\mathcal{V}') \\
\text{Prob}(X_T = \mathcal{V}\mathcal{V}') \\
\text{Prob}(X_T = \mathcal{I}'\mathcal{I}') \\
\text{Prob}(X_T = \mathcal{V}'\mathcal{I}') \\
\text{Prob}(X_T = \mathcal{I}'\mathcal{V}') \\
\text{Prob}(X_T = \mathcal{V}'\mathcal{V}')
\end{bmatrix} = H_T \boldsymbol{e}_1. \tag{A2}
$$

By using (A2), the state probabilities are given explicitly by

$$\text{Prob}(X_T = N) = \prod_{t=0}^{T} s_N(t), \tag{A3a}$$

$$\text{Prob}(X_T = \mathcal{I}) = s_{\mathcal{I}N}(T) \prod_{t=0}^{T-1} s_N(t), \tag{A3b}$$

$$\text{Prob}(X_T = \mathcal{V}) = s_{\mathcal{V}N}(T) \prod_{t=0}^{T-1} s_N(t), \tag{A3c}$$

$$\text{Prob}(X_T = \mathcal{I}') = \sum_{t=0}^{T-1} \left(\prod_{\sigma=t+2}^{T} s_{\mathcal{I}'}(\sigma) \right) s_{\mathcal{I}N}(t) \prod_{\ell=0}^{t-1} s_N(\ell), \tag{A3d}$$

$$\text{Prob}(X_T = \mathcal{V}') = \sum_{t=0}^{T-1} \left(\prod_{\sigma=t+2}^{T} s_{\mathcal{V}'}(\sigma) \right) s_{\mathcal{V}N}(t) \prod_{\ell=0}^{t-1} s_N(\ell), \tag{A3e}$$

$$\text{Prob}(X_T = \mathcal{I}\mathcal{I}') = s_{\mathcal{I}\mathcal{I}'}(T) \sum_{t=0}^{T-2} \left(\prod_{\sigma=t+2}^{T-1} s_{\mathcal{I}'}(\sigma) \right) s_{\mathcal{I}N}(t) \prod_{\ell=0}^{t-1} s_N(\ell), \tag{A3f}$$

$$\text{Prob}(X_T = \mathcal{V}\mathcal{I}') = s_{\mathcal{V}\mathcal{I}'}(T) \sum_{t=0}^{T-2} \left(\prod_{\sigma=t+2}^{T-1} s_{\mathcal{I}'}(\sigma) \right) s_{\mathcal{I}N}(t) \prod_{\ell=0}^{t-1} s_N(\ell), \tag{A3g}$$

$$\text{Prob}(X_T = \mathcal{I}\mathcal{V}') = s_{\mathcal{I}\mathcal{V}'}(T) \sum_{t=0}^{T-2} \left(\prod_{\sigma=t+2}^{T-1} s_{\mathcal{V}'}(\sigma) \right) s_{\mathcal{V}N}(t) \prod_{\ell=0}^{t-1} s_N(\ell), \tag{A3h}$$

$$\text{Prob}(X_T = \mathcal{V}\mathcal{V}') = s_{\mathcal{V}\mathcal{V}'}(T) \sum_{t=0}^{T-2} \left(\prod_{\sigma=t+2}^{T-1} s_{\mathcal{V}'}(\sigma) \right) s_{\mathcal{V}N}(t) \prod_{\ell=0}^{t-1} s_N(\ell), \tag{A3i}$$

$$\text{Prob}(X_T = \mathcal{I}'\mathcal{I}') = \sum_{t=2}^{T-1} s_{\mathcal{I}\mathcal{I}'}(t) \sum_{i=0}^{t-2} \left(\prod_{\sigma=i+2}^{t-1} s_{\mathcal{I}'}(\sigma) \right) s_{\mathcal{I}N}(i) \prod_{\ell=0}^{i-1} s_N(\ell), \tag{A3j}$$

$$\text{Prob}(X_T = \mathcal{V}'\mathcal{I}') = \sum_{t=2}^{T-1} s_{\mathcal{V}\mathcal{I}'}(t) \sum_{i=0}^{t-2} \left(\prod_{\sigma=i+2}^{t-1} s_{\mathcal{I}'}(\sigma) \right) s_{\mathcal{I}N}(i) \prod_{\ell=0}^{i-1} s_N(\ell), \tag{A3k}$$

$$\text{Prob}(X_T = \mathcal{I}'\mathcal{V}') = \sum_{t=2}^{T-1} s_{\mathcal{I}\mathcal{V}'}(t) \sum_{i=0}^{t-2} \left(\prod_{\sigma=i+2}^{t-1} s_{\mathcal{V}'}(\sigma) \right) s_{\mathcal{V}N}(i) \prod_{\ell=0}^{i-1} s_N(\ell), \tag{A3l}$$

$$\text{Prob}(X_T = \mathcal{V}'\mathcal{V}') = \sum_{t=2}^{T-1} s_{\mathcal{V}\mathcal{V}'}(t) \sum_{i=0}^{t-2} \left(\prod_{\sigma=i+2}^{t-1} s_{\mathcal{V}'}(\sigma) \right) s_{\mathcal{V}N}(i) \prod_{\ell=0}^{i-1} s_N(\ell), \tag{A3m}$$

where we adopt the convention that if the top index of a product is greater than the bottom index, the result is the empty product, whose value is 1.

B Additional data visualizations and models

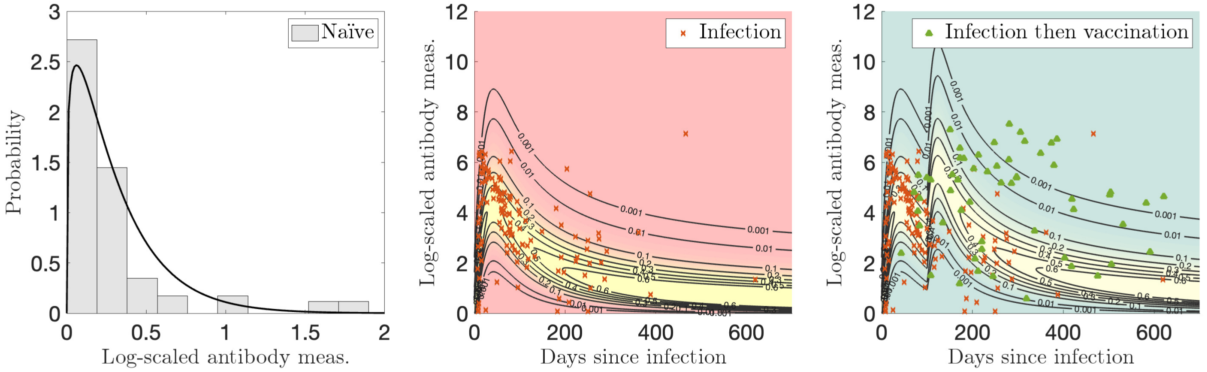

Figure B1: Log-transformed antibody measurements from the naïve, infected (I), and infected then vaccinated (VI) populations from Dataset 1 with corresponding probability models.

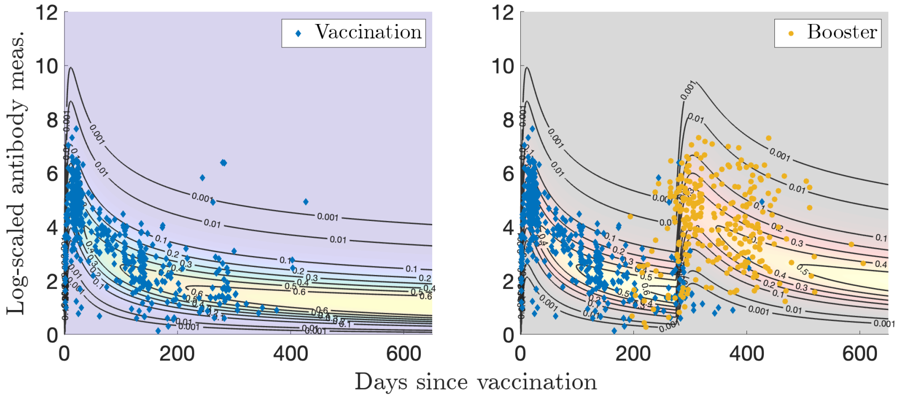

Figure B2: Log-transformed antibody measurements from the vaccinated (V) and boosted (VV) populations from Dataset 1 with corresponding probability models.

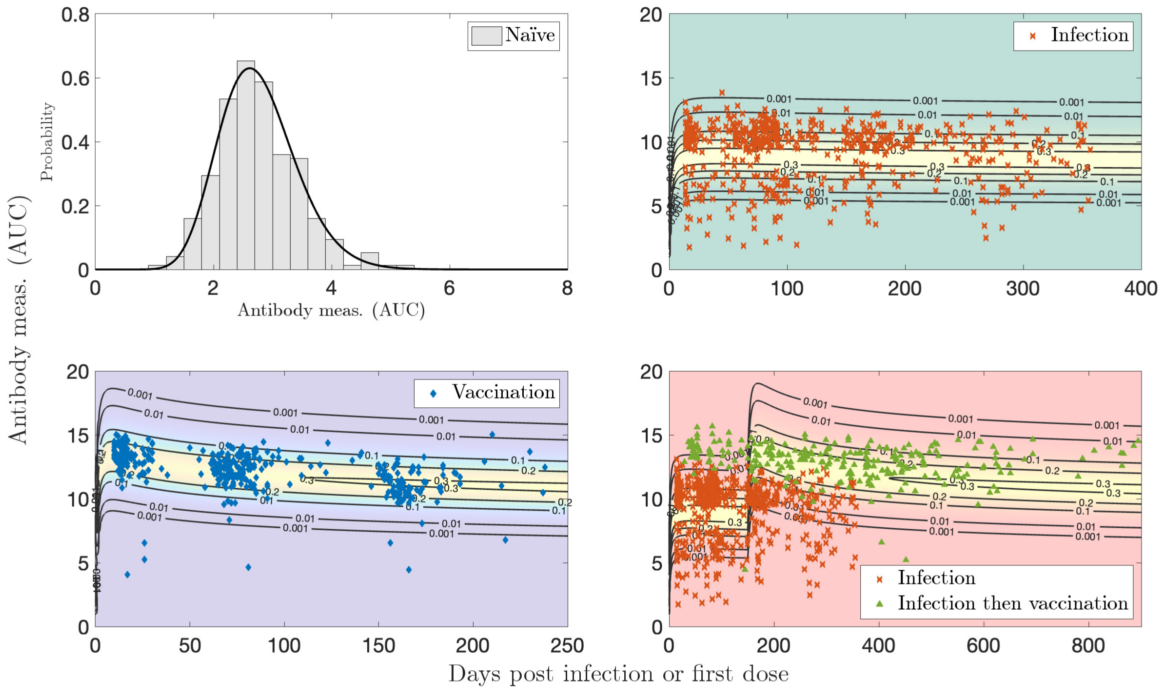

Figure B3: Log-transformed antibody measurements from the naïve (synthetic), infected (I), vaccinated (V), and infected then vaccinated (VI) populations from Dataset 2 with corresponding probability models. Measurements with recorded monotonically decreasing OD values have been AUC-extrapolated using the next titration level.
